# Ring-string traction for successful endoscopic clip closure to treat delayed perforation of the ulcer bed after colorectal endoscopic submucosal dissection

**DOI:** 10.1055/a-2279-6863

**Published:** 2024-04-03

**Authors:** Rie Tsukida, Yoshihiro Kishida, Kenichiro Imai, Kazunori Takada, Sayo Ito, Kinichi Hotta, Hiroyuki Ono

**Affiliations:** 1 38471 Division of Endoscopy, Shizuoka Cancer Center, Shizuoka, Japan

Endoscopic closure of delayed perforations after colorectal endoscopic submucosal dissection (ESD) is challenging due to the fragility of the ulcer bed and the stiffness of the surrounding tissue 1 2 3 4 . The case presented here illustrates closure of an ulcer bed with delayed perforation after ESD by endoscopic mucosa-to-mucosa closure using a ring-string traction ( Fig. 1 ; Video 1 ).

**Fig. 1:**
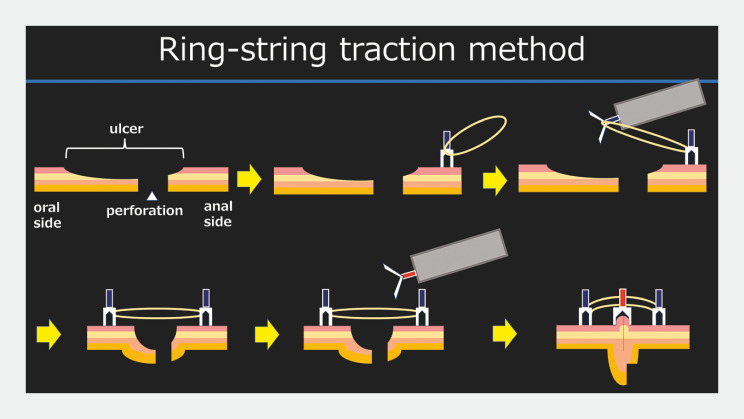
Schematic of ring-string traction method for endoscopic mucosa-to-mucosa closure of ulcer bed following delayed perforation after colorectal endoscopic submucosal dissection (ESD).

Ring-string traction facilitates endoscopic clip closure to treat delayed perforation of the ulcer bed after colorectal endoscopic submucosal dissection.Video 1

A 47-year-old woman underwent ESD for a laterally spreading tumor (0-Is+IIa, 60×53mm) in the transverse colon ( Fig. 2 **a** , **b** ). En bloc resection was achieved without any adverse event during the ESD procedure ( Fig. 2 **c** , **d** ). Fever and abdominal pain occurred 40 hours later. Delayed perforation was diagnosed on the basis of free air shown on computed tomography. During emergency endoscopy, a pinhole-sized mural defect in the ulcer was confirmed ( Fig. 3 **a** ). We abandoned closure by clips because of the fragile ulcer bed and the thickly edematous surrounding mucosa; instead we decided to use a ring-string traction technique. To do this, a hand-made small (diameter 1 cm) ring of dental floss (string) was grasped with a retractable clip, drawn through the scope, and fixed to the normal mucosa on the anal side of the ulcer. Next, the ring-string was pulled toward the oral side of the ulcer with another retractable clip and likewise fixed to the normal mucosa. With two ring-string tractions, the ulcer became narrow ( Fig. 3 **b** ). Complete clip closure of the ulcer was then achieved ( Fig. 3 **c** ). The patient recovered with antimicrobial treatment and was discharged on the 7th day.

**Fig. 2:**
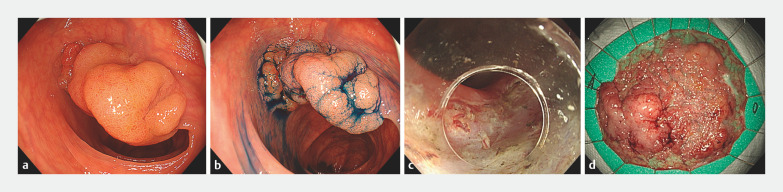
Endoscopic images of lesion and ESD procedure. **a** Laterally spreading tumor (LST) in the transverse colon. **b** Chromoendoscopic view. **c** No perforation in the ulcer bed. **d** ESD was achieved without adverse events.

**Fig. 3:**
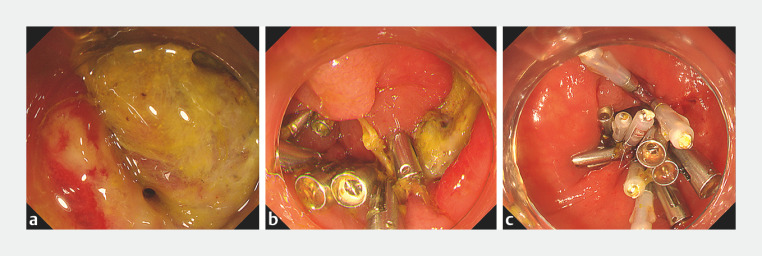
Endoscopic images of delayed perforation and closure using ring-string traction. **a** Delayed perforation (40 hours after ESD) in the ulcer bed. **b** With traction using a ring-string fixed with retractable clips, the normal mucosa on the anal and the oral sides of the ulcer were brought closer together. **c** Complete ulcer closure was achieved with clips.

In this case, ring-string traction showed two advantages. First, delivery of the device through the scope enabled an immediate closure operation. Second, the technique is convenient and cost-effective because it does not require specific devices. This case suggests that ring-string traction would be useful for endoscopic closure after delayed perforation of the ulcer bed after ESD, and could help to avoid emergent surgery.

Endoscopy_UCTN_Code_CPL_1AJ_2AD

## Notes

Endoscopy E-Videos https://eref.thieme.de/e-videos
*E-Videos* is an open access online section of the journal *Endoscopy* , reporting on interesting cases and new techniques in gastroenterological endoscopy. All papers include a high-quality video and are published with a Creative Commons CC-BY license. Endoscopy E-Videos qualify for HINARI discounts and waivers and eligibility is automatically checked during the submission process. We grant 100% waivers to articles whose corresponding authors are based in Group A countries and 50% waivers to those who are based in Group B countries as classified by Research4Life (see: https://www.research4life.org/access/eligibility/ ). This section has its own submission website at https://mc.manuscriptcentral.com/e-videos .
